# ‘I live in extremes’: A qualitative investigation of Autistic adults’ experiences of inertial rest and motion

**DOI:** 10.1177/13623613231198916

**Published:** 2023-09-30

**Authors:** Hannah Rapaport, Hayley Clapham, Jon Adams, Wenn Lawson, Kaśka Porayska-Pomsta, Elizabeth Pellicano

**Affiliations:** 1 Macquarie School of Education, Macquarie University, Sydney, Australia; 2 UCL Institute of Education, University College London, UK; 3 Department of Clinical, Educational and Health Psychology, University College London, UK

**Keywords:** attention, flow, monotropism, quality of life, wellbeing

## Abstract

‘Autistic inertia’ is part of the Autistic community’s everyday lexicon, yet the condition has received scant attention in the academic literature. The little attention it has received has focused on the disabling impact of ‘inertial rest’ (difficulty initiating tasks), with minimal exploration of the potential benefits of inertial motion (difficulty stopping tasks). Here, we sought to investigate Autistic people’s phenomenological experiences of inertial rest and motion and to identify factors that might moderate Autistic inertia. Autistic and non-Autistic researchers conducted semi-structured interviews with 24 Autistic adults. We analysed the data using reflexive thematic analysis, adopting an inductive approach within an essentialist framework. Interviewees spoke in-depth of their inertial ‘difficulties moving from one state to another’ and described how these challenges pervade ‘every single day’. While inertia was described as ‘the single most disabling part of being Autistic’, interviewees also described ways to respond to inertia, including taking pleasure in states of flow, whereby they could become completely absorbed in a task. While inertial difficulties are pervasive, our findings reveal the extreme nature of these task-switching difficulties for Autistic people. Our findings also contribute to a more holistic characterisation of inertia as both a disabling and enabling condition.

**Lay Abstract:**

‘Autistic inertia’ is a term used by Autistic people to refer to difficulties with starting and stopping tasks. However, there has not been much research on Autistic inertia. The research that is available on Autistic inertia has mostly focused on the negative aspects of inertia, rather than on the possible benefits of needing to continue tasks. In this research, we wanted to understand more about Autistic people’s experiences of inertia and to work out what things might influence these experiences. Autistic and non-Autistic researchers spoke in-depth to 24 Autistic adults. We identified four key ideas from people’s responses. Autistic people spoke about their inertial ‘difficulties moving from one state to another’ and described how these challenges affected them ‘every single day’. While they experienced inertia as ‘the single most disabling part of being Autistic’, people also described the positive aspects of inertia, including the joy they felt when completely immersed in a task. Our Autistic participants emphasised that inertial difficulties are experienced by everyone, the intensity of these task-switching difficulties might be especially challenging for Autistic people. Our findings also reveal how Autistic inertia can be seen both as a disabling and as an enabling condition.

Under Newton’s ‘law of inertia’, an object at rest stays at rest, and an object in motion stays in motion at a constant velocity, unless that state is changed by a force (Newton, 1687). The term ‘inertia’ was adopted by the Autistic^1^ community in the 1990s to refer to the seemingly common experience of remaining in a mental and physical state of rest or motion until intervention (Buckle et al., 2021; Dekker, 1999). Although ‘Autistic inertia’ is part of the Autistic community’s everyday lexicon, it has received scant attention in the academic literature. Here, we sought to further our understanding of Autistic people’s experiences of inertia.

Inertia can be understood as two converse states: ‘inertial rest’ and ‘inertial motion’. Regarding the former, Autistic people describe becoming ‘stuck’ or ‘frozen’ while attempting to complete everyday tasks. For example, Autistic scholar and advocate Martijn Dekker notes that ‘the inertial person has problems getting started with things, such as doing housework, filling in tax forms . . . even if the motivation to do it is present’ (Dekker, 1999, p. 8). Indeed, Autistic people have described experiences of inertia as being their ‘single biggest problem’ (Buckle et al., 2021).

Autistic people also describe, however, apparent advantages of inertia – especially the experience of being so deeply immersed in an activity or a thought that they cannot, or do not want to, stop. Thus, people can remain in a state of ‘inertial motion’ until interrupted. This Autistic tendency to focus intensely on tasks, topics or objects of interest is described negatively in the clinical literature as ‘restricted, repetitive patterns of behaviour, interests or activities’ that are ‘abnormal in intensity or focus’, such as an ‘adult spending hours writing out timetables’ (American Psychiatric Association [APA], 2013, pp. 31, 50, 54). Yet Autistic people themselves often describe these periods of hyper-focus as achieving the desirable optimal ‘flow state’ (Boren, 2020; Buckle et al., 2021; McDonnell & Milton, 2014; Sparrow, 2016) – an exhilarating state of consciousness, whereby a person becomes completely absorbed in an activity (Csikszentmihalyi, 2000). Indeed, one Autistic man described being so ‘focused on something’ that he was ‘not aware of how much time’s passed’ (Buckle et al., 2021, p. 7). Anecdotal reports further suggest that experiences of ‘flow’ or ‘inertial motion’ can give rise to periods of great productivity and ‘tap into . . . areas of creativity’ (Sparrow, 2016).

Although Autistic people have been writing about their experiences of inertia for many years, the topic has been overlooked by autism researchers. The first scientific recognition of Autistic inertia seems to emerge in descriptions of what clinicians instead refer to as ‘Autistic catatonia’ or ‘autism-related catatonia’ (Hare & Malone, 2004; Shah, 2019a; Wing & Shah, 2000). According to the *Diagnostic and Statistical Manual of Mental Disorders* (5th ed.; DSM-5; APA, 2013), catatonia is a marked psychomotor disturbance that can occur in the context of several conditions, including autism. Consistent with the inertia metaphor, the DSM-5 describes the clinical presentation of catatonia as ranging from ‘marked unresponsiveness to marked agitation’ (APA, 2013, p. 119). Indeed, many of the characteristics of Autistic catatonia and Autistic inertia appear, at face value, to be shared, including periods of ‘freezing’ or becoming physically or mentally ‘stuck’, dependence on verbal or physical prompts for functioning, and difficulty initiating a movement or stopping a movement once started (APA, 2013, p. 55; Shah, 2019a).

Unlike Autistic inertia, however, which is described by Autistic people as being both disabling (Phung et al., 2021; Welch et al., 2019, 2021) and advantageous (Buckle et al., 2021; Dekker, 1999), Autistic catatonia is described solely as a disabling condition, whereby Autistic people either become stuck while attempting to complete activities or persevere to the point of exhaustion (Shah, 2019c). Furthermore, the concept of Autistic catatonia appears to be largely informed by clinical observation rather than by firsthand accounts (Shah, 2019b). As such, the literature on Autistic catatonia may well miss important details regarding Autistic people’s lived experiences, including its physical sensations, psychological impact, contextual nuances and impact on people’s everyday lives.

One of the first studies to address the lack of firsthand accounts of Autistic inertia did so by conducting online and in-person focus groups with Autistic people (Buckle et al., 2021). Participants were asked about: (1) times when they experienced difficulties doing things; (2) factors that made doing these things harder or easier; (3) what it feels like to be ‘stuck’ and (4) the impact of these experiences on their everyday lives. Participants described difficulties with executing plans and initiating action and reliance on prompting by another person to start or sustain action. Stress and poor mental health were reported to exacerbate inertial difficulties. While some described the advantages of becoming ‘totally immersed in some things’ (p. 7), most responses were related to the debilitating aspects of Autistic inertia. This focus is unsurprising, given that the authors themselves note that the ‘questions were oriented around difficulty doing things’ (p. 4). Other studies of inertia have likewise concentrated on the debilitating impact of feeling stuck, with no mention of the potential positive side of inertial motion (Phung et al., 2021; Welch et al., 2019, 2021). Thus, Autistic inertia, as both a disabling and enabling condition, is far from being fully understood.

This study, therefore, extended Buckle et al.’s (2021) initial work to understand the full range of Autistic adults’ inertial experiences. Specifically, we used in-depth semi-structured interviews to investigate Autistic people’s experiences of inertial rest and motion, and the factors that might moderate Autistic inertia.

## Method

### Participants

We advertised the study via social media with a video and flyer. Eligible participants were (1) fluent in English; (2) living in Australia; (3) aged 18 years or older and (4) clinically diagnosed or self-identified as Autistic (the latter to account for the often-significant barriers in gaining a clinical diagnosis; Lai & Baron-Cohen, 2015). We intentionally avoided using ‘Autistic inertia’ in our recruitment materials and interview questions (detailed below) so that we stayed close to participants’ reports of everyday phenomena, rather than imposing specific terms. Of the 53 expressions of interest, 29 were excluded as they did not (1) live in Australia (*n* = 9); (2) complete the consent form (*n* = 9); (3) confirm an interview appointment (*n* = 5); (4) return email interview responses (*n* = 4) or (5) attend the interview (*n* = 2).

The remaining 24 participants were aged 19–71 years (M = 44.3; SD = 16.4). Eleven (46%) identified as female, seven (29%) as male, four (17%) as non-binary/genderqueer, and two (8%) as ‘other’. Participants reported themselves to be predominantly white, highly educated and currently employed (see Table 1). All had an independent clinical diagnosis of autism (M age of diagnosis = 37.3 years, SD = 17.3; range = 3–61 years) from a clinical psychologist (*n* = 17; 71%), a team of health professionals (*n* = 4; 17%), psychiatrist (*n* = 2; 8%) or an autism specialist (*n* = 1; 4%). Most reported co-occurring diagnoses – especially depression, anxiety disorder and attention-deficit/hyperactivity disorder (see Table 1).

**Table 1.** Interviewee characteristics.

Demographic information	M or *n*	SD or %	Min	Max
Age (years)	44.3	16.4	19	71
Age at autism diagnosis (years)	37.3	17.3	3	61
Gender				
Women (including trans women)	11	46%		
Men (including trans men)	7	29%		
Non-binary	4	17%		
Other^a^	2	8%		
Predominant racial/ethnic background				
Arab	1	4%		
Mixed (White/Middle Eastern)	1	4%		
South-East Asian	1	4%		
White European	18	75%		
White Other	3	13%		
Usual communication mode				
Spoken	22	92%		
Partner-assisted typing	1	4%		
Sign language or written communication	1	4%		
Living arrangements				
Alone	4	17%		
With a partner only	6	25%		
With partner and children	8	33%		
With parents	4	17%		
With other relatives	1	4%		
With friends	1	4%		
Highest qualification				
Primary school	1	4%		
High school	2	8%		
Vocational training	5	21%		
University degree	16	67%		
Occupational status				
Full-time employed	8	36%		
Full-time employed and studying	2	8%		
Part-time employed	6	24%		
Part-time employed and studying	4	16%		
Unemployed and studying	1	4%		
Unemployed and not studying	3	12%		
Co-occurring conditions^b^				
ADHD	12	50%		
Anxiety disorders	17	71%		
Bipolar disorder	2	8%		
Cancer	2	8%		
Chronic fatigue	2	8%		
Depression	18	75%		
Dyslexia	1	4%		
Dyspraxia	3	13%		
Eating disorders	5	21%		
Ehlers Danlos Syndrome	4	17%		
Epilepsy	2	8%		
Gastrointestinal issues	9	38%		
Hypertension (high blood pressure)	7	29%		
Intellectual disability	1	4%		
Immune condition	3	13%		
OCD	2	8%		
Obesity	5	21%		
Personality disorder	2	8%		
PTSD and/or C-PTSD	9	38%		
Schizophrenia disorders	3	13%		
Sleep disorders	12	50%		
Stroke	2	8%		

ADHD: attention-deficit/hyperactivity disorder; OCD: obsessive-compulsive disorder; PTSD: post-traumatic stress disorder; C-PTSD: complex post-traumatic stress disorder.

a Gender was self-described as ‘Autistic’ by one participant, and ‘demi-girl’ by another.

b Participants could select all options that applied to them. Percentages, therefore, do not add to 100.

### Procedure

Macquarie University Human Research Ethics Committee (Project ID: 520211064335595) approved all study procedures. All participants provided written informed consent before taking part.

Participants provided their demographic information via an online questionnaire (LimeSurvey, Version 5.3.32, LimeSurvey GmbH, Hamburg, Germany). They then participated in individual, semi-structured interviews via their preferred means of communication, including Zoom (*n* = 18), email (*n* = 5) or phone (*n* = 1) between January and March 2022. Spoken interviews were conducted by either an Autistic (*n* = 9) or a non-Autistic researcher (*n* = 10), both employed on the project.

Participants answered open-ended questions about their experiences of starting and stopping tasks, including how they felt during these states, the frequency and duration of these states, their perceived causes, things that help them to move out of these states and the impact of these states on their everyday lives (see Supplementary Materials for interview schedule). Primary interview questions were emailed to participants before the interview.

Spoken and written interviews ranged in length between 34 and 107 min (Mdn = 58; SD = 19.7) or 637 and 3800 words (Mdn = 1860; SD = 1262), respectively. Spoken interview audio recordings were transcribed verbatim by a professional service. Participants reviewed their de-identified transcripts and received an AUD$25 voucher for their time. One participant redacted sensitive information from their transcript.

### Data analysis

Our analysis was informed by our collective experience and training in psychology (H.R., H.C., W.L. and E.P.), education (E.P., K.P.-P.), computer science (K.P.-P.), creativity and mental health (J.A.) and by positionalities as Autistic people (H.C., J.A. and W.L.). We followed Braun and Clarke’s (2006, 2019) reflexive thematic analytic method within an essentialist framework, whereby we aimed to report participants’ experienced realities. We used an inductive (‘bottom-up’) approach to identify key themes, that is, without integrating the themes within any pre-existing coding schemes or preconceptions of the researchers.

The analytic process began during data collection, during which we attempted to minimise the influence of our preconceptions by giving participants space to share their views, ideas and experiences and being guided by what we interpreted to be meaningful to the interviewee. During this time, H.R. kept informal memos and had debriefs with E.P. and H.C. to discuss analytic ‘noticings’. All the authors then met regularly to discuss patterns in the data and potential codes (and eventually, themes), while at the same time reflecting on our assumptions and how they might be shaping our interpretation of interviewees’ responses. Following these discussions, one researcher (H.R.) immersed themselves in the data, reading and re-reading all transcripts closely, taking reflexive notes on striking and recurring observations and applying codes to each transcript (managed in NVivo, version 1.6.1). Codes were clustered to identify potential themes and subthemes. H.R. then produced a draft thematic map. Relevant data were collated under each theme and subtheme, focusing on semantic features of the data (staying close to the interviewees’ language). The draft analysis was revised initially by E.P., and then multiple times with the broader team, where the themes and subthemes were reflexively refined (Braun & Clarke, 2019), with consideration of researcher assumptions, community input and insights from the analytic process. We followed the Standards for Reporting Qualitative Research guidelines (O’Brien et al., 2014).

### Community involvement

Consistent with best-practice participatory approaches (Fletcher-Watson et al., 2019; Nicolaidis et al., 2011; Pellicano & Stears, 2011), Autistic scholars and advocates (J.A., H.C. and W.L.) were actively involved in every stage of the research process from project inception (W.L. was named on the grant application) to choosing the research methods, designing the study and the interview schedule, conducting interviews (H.C.), analysing and interpreting the findings and commenting on the draft manuscript. We met regularly over Zoom, which resulted in collaborative decisions that improved the relevance, clarity and accessibility of materials and the nature and content of the interview itself. During analysis, all the authors met regularly to reflect thoroughly upon individual transcripts and the subsequent thematic map, which led to changes in the thematic structure and individual theme/subtheme labels.

## Results

**Figure 1.**
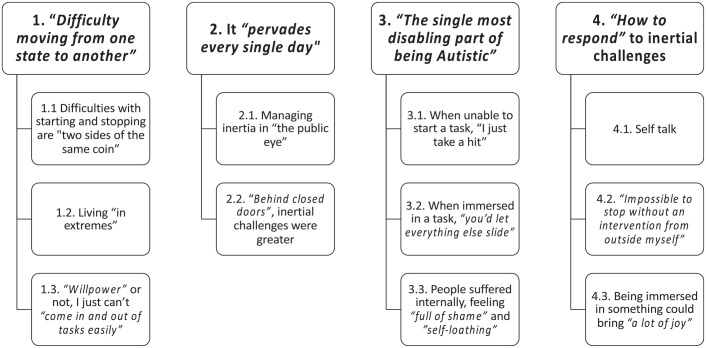
Participants’ perspectives on their starting and stopping difficulties on everyday tasks: themes and subthemes.

We identified four themes, summarised in Figure 1 and described below. Themes are listed under subheadings and subthemes are italicised. We use illustrative quotes throughout (attributed via participant ID numbers in square brackets).

### Theme 1: ‘Difficulty moving from one state to another’

Participants felt that their *starting and stopping difficulties are* ‘*two sides of the same coin*’ (subtheme 1.1) and ‘very interlinked’ [P115]: ‘The things that affect my ability to start . . . also affect my ability to stop’ [P115]. Specifically, participants felt that their starting and stopping difficulties were underpinned by ‘a struggle with change’ [P109] – particularly ‘when something gets changed unexpectedly’ [P117]. For example, when – during a doctor’s appointment – one participant’s ‘expectation of what was supposed to happen, didn’t’ [P117], they were left feeling paralysed: ‘I couldn’t move . . . I couldn’t even speak’. Conversely, a violated expectation during a shopping routine left another participant stuck in motion:

[I] was about to walk home and the handle fell off the bag . . . I couldn’t actually change at that point and stop and grab another bag. I actually had to complete the pattern of getting out [of the store]. [P143]

In these situations, participants reported a ‘tendency to continue on the same trajectory’ rather than attempting to ‘dynamically deal with people or circumstances that are unpredictable’ [P143]. One participant remarked, ‘If there’s a small change in an expectation, I’ve got no idea how to negotiate that’ [P117]. Thus, remaining in the same state seemed to be preferable: ‘It’s a relief to be in a regulated state and I want to stay that way. It’s really hard to transition away from that’ [P113].

Participants also reported that they could not ‘just come in and out of tasks easily’ [P115], and to do so was ‘so mentally draining for me’ [P115]. They described how ‘changing track takes a lot of time. It takes energy . . . [and] will leave me exhausted’ [P143], in part because ‘any new task requires that I switch off the first and re-factor things again’ [P123]. One participant drew on Newton’s law of inertia to describe the energy costs involved in transitioning between tasks:

When something is stopped, it requires a certain amount of energy to get it started. And then when it’s started, it requires a certain amount of energy to get it stopped again . . . even though it’s talking about physics, it explains almost exactly my experience of tasks. [P142]

Owing to their inertial difficulties with ‘moving from one state to another’ [P113], participants described *living ‘in extremes’* (subtheme 1.2): ‘I have two modes: I have “can’t stop”, and “can’t start”, and there’s nothing in between. I’m either go, go, go or can’t move’ [P125]. Regarding the ‘can’t start’ mode, participants described ‘difficulties with initiation of any activity, be it physical, like brushing my teeth, or mental such as typing to talk or writing an essay’ [P123]. The metaphors of a mental wall, barrier, block or obstacle were frequently used to describe how participants felt when they could not get started on a task. Participants also described feeling ‘a sense of heaviness’ [P113], like ‘lead’ [P125; P117] and spoke of ‘challenges in body awareness – a fragmented body map in the sense that I can’t always feel parts of the body, so it becomes very hard to co-ordinate my body parts to do the movements [to initiate tasks]’ [P123].

Regarding the other extreme – the ‘can’t stop’ mode – participants spoke of becoming so engrossed in a task that they were either unable or unwilling to stop or switch tasks, irrespective of the environmental demands. Difficulties with stopping were reported to affect cognitive tasks, such as ‘reading a book’ [P111], and physical activities, such as ‘when I stim – that is, do fidgety things compulsively’ [P123]. Participants described their difficulty with stopping tasks as due to ‘being obsessive (like, I had to research a lot of things)’ [P134] or because they were in a state of hyper-focus: ‘If it’s something I’m enjoying or I’m fixated on, I can’t stop. Not easily’ [P109]:

Particularly if I’m studying or learning about something new . . . and it really taps into my passions – once I get started, stopping is not an option. It’s like riding a bike down a very steep hill, you just keep going. Not eating, not drinking, trying to avoid going to the toilet, overriding everything, not being aware of time passing. [P105]

Regarding both extremes, participants reported that *‘willpower’ or not, they just can’t ‘come in and out of tasks easily’* (subtheme 1.3). For example, participants would ‘avoid and struggle to start’ tasks that were ‘not in my interest zone’ [P115]: ‘If I find the task boring, or repetitive, or mundane, or something like that, then I find it difficult to get started on it’ [P124]. These ‘non-preferred tasks’ would be ‘postponed till the next week’ [P125], or participants would ‘avoid them as much as possible’ [P134] while prioritising ‘other things, like maybe work or seeing friends . . . that are more motivating to do’ [P115]. However, participants also described experiences of ‘wanting to do it [the task], and literally having no barriers, but just not being able to do it’ [P131] – ‘I simply get stuck’ [P136].

### Theme 2: It ‘pervades every single day’

Starting and stopping difficulties were described as something that ‘pervades every single day’ [P103] and could occur ‘all the time, with almost all tasks’ [P123]. The context, however, seemed to moderate participants’ inertial experiences. For example, participants found *managing inertia in ‘the public eye’* (subtheme 2.1) to be easier. In public settings, there were more likely to be external prompts – a ‘hard deadline’ [P115] from a boss or a ‘supervisor [saying] . . . “you can work on this task”’ [P139]. Some felt ‘really fortunate in that I have someone who can offer me some support . . . at work’ [P139] and someone ‘in school . . . [who] helped me break down the tasks so that there were fewer barriers’ [P131], while others felt that, at times, ‘scaffolding structure . . . will add pressure’ [P117], resulting ‘in burnout, time off work and changing jobs’ [P115].

Nevertheless, in contexts where there were less likely to be external prompts – ‘around the home’ [P106] and *‘behind closed doors’, inertial challenges were greater* (subtheme 2.2). Participants reported greater initiation challenges with ‘cooking, cleaning, housework’ [P143], ‘my study life, or exercise, or dieting . . . keeping up with self-care, and bills’ [P115]. Some found that ‘working from home I think is harder’ than working in a public workspace due to the absence of ‘someone else there to prompt it, just to say, let’s get started on this today’ [P142]: ‘I will often find myself sitting at the computer and just be absolutely stuck, and just be sitting here staring at the screen going “I don’t know what to do”’ [P142]. Domestic chores seemed to be particularly difficult to get started on without guidance and prompting:

I have, as an adult, ongoing issues . . . like keeping my house clean, keeping on top of chores, and things like being organised and making appointments. And I feel like a lot of it is about feeling overwhelmed in terms of steps. And I have a lot of difficulty determining what’s the first step when I have a task. So, something like cleaning a house, for example, even if I break that down to cleaning the kitchen, I’m still where do I start, do I vacuum, should I mop, should I do the dishes first, what dishes do I do first and where do we keep the things for it? I just find I get really overwhelmed really easily. [P139]

Those who seemed better able to manage their inertial challenges in private spaces had support at home – from a partner, occupational therapist, social worker or home organiser:

I did have a support worker who was really good at prompting . . . always being really supportive and, ‘It’s okay if you can’t do it. If you need to take a break, whatever, let me know what you need, let me know what I can do to help’. It was a really good balance of being directive enough and giving me the guidance that I needed, without me feeling pressured. [P142]

### Theme 3: ‘The single most disabling part of being Autistic’

Participants felt that their task-switching difficulties are ‘very human problems’ [P115] but differentiated the Autistic experience in terms of the severity of these difficulties: ‘People like me really struggle to a higher level with those things to the point where it does impact all aspects of our life’ [P115]. Indeed, they felt the inertial difficulties could be ‘the single most disabling part of being Autistic . . . it’s a daily struggle’ [P142]:

Can’t start and then can’t stop. If I was a car, I’d be useless, you know? You turn the ignition on and it doesn’t go, and then when it finally does go, the brakes don’t work. It’s been a hindrance in my life, not a positive. [P109]

Participants reported that, *when unable to start a task, ‘I just take a hit’* (subtheme 3.1). Such negative impacts could be far-reaching, affecting relationships, study, work, parenting and their ability to be ‘a productive adult in society’ [P115]. Relationships could ‘become strained because I’m perceived as lazy’ [P131]. It could be hurtful when neurotypical people lacked understanding of Autistic people’s task initiation challenges, ‘because I’m doing my best’ [P103]:

It is a source of argument between myself and my wife. Because she thinks that it’s actually easy for me to switch from doing one thing to another because everyone can do that. For me, that’s actually quite hard. [P143]

Participants also reported that, *when immersed in a task, ‘you let everything else slide’* (subtheme 3.2):

It’s a serious struggle. It can be great to get totally engrossed in something . . . but completely impractical. And suddenly I’ve missed several writing deadlines I was hoping to meet, and I’ve fallen behind in my university reading, and things are generally looking pretty bleak. [P136]

Participants also felt ‘like a burden to everyone’ [P119] due to their reliance on others to ‘bring me out of it’ [P139]. Without such prompting, however, participants could ‘enter a kind of hyper-focus state’ that was so intense that they could ‘completely forget to eat, drink’ [P115], ‘go to the bathroom, have a stretch’ [P139] and to get ‘sufficient sleep’ [P113]. As one participant put it, ‘It’s not good . . . there’s no balance’ [P131].

Participants could also ‘have a high tolerance level for pain, so it’s also difficult to stop – e.g. brushing my teeth even when I do it too long or too hard . . . I keep going until my gum bleeds’ [P123]. While stimming was initially calming, doing it ‘for too long’ could be ‘physically unpleasant’ [P123]. One participant described that ‘when I stim, the degree of satisfaction/enjoyment levels off and is replaced by just going through the motions, e.g. when I tap on a surface or keep going with typing to talk even when the person has left’ [P123]. ‘The not stopping’ could also leave participants feeling ‘mentally exhausted’, ‘really stressed that I don’t know where all the time’s gone’ and ‘because I can’t stop, I’m more and more burnt out’ [P125]: ‘You start wearing yourself out’ [P124].

Inertial challenges were, ‘to the outside world’ [P103], often hidden: ‘I find that these challenges, arising from my Autistic differences, are invisible, although their impact is pervasive’ [P123]. As such, *participants suffered internally, feeling ‘full of shame’ and ‘self-loathing’* (subtheme 3.3): ‘My inability to do things makes me feel lesser than others’ [P127]. At times, they would be ‘mentally telling myself off’ [P139], saying ‘Why can’t you just get on with it!’ [P136] and ‘What’s wrong with me?’ [P128]. Participants felt as though they had ‘seriously under-achieved’ [P105] and ‘haven’t lived up to my full potential’ [P109], all ‘because I’m not nearly as productive as I should be, I’m expected to be and would like to be’ [P131].

Nevertheless, in the face of these ‘more negative thoughts about myself’ [P136], participants also showed great strength: ‘I’ve tried to be a little bit more kind to myself’ [P139]. They ‘developed management strategies . . . like I would tell people to be patient with me’ [P123]. ‘Getting a diagnosis [of autism]’ too could be ‘a huge life-changing thing’, as it could give ‘context to my brain and identity [and] lifted a lifetime of shame and self-loathing’ [P115]. It also helped participants to understand their inertia as part of the Autistic experience: ‘I’m not going to beat myself up anymore, I’m just going to just accept that this is a part of how my brain works, and when it’s working it works fantastically, and I have to be grateful for that’ [P115].

### Theme 4: ‘How to respond’ to inertial challenges

In response to inertial challenges, some would remain stuck until there was some form of internal ‘cueing’ [P123] or *‘self-talk’* (subtheme 4.1) – a ‘little voice’ that ‘says “you’re going to keep going”, or the voice that says “you need to stop”’ [P124]. However, some found it ‘*impossible to stop without an intervention from outside myself*’ (subtheme 4.2), such as prompting from assistive technology, ‘a family member, a dog’ [P136], a colleague or a support worker:

I need prompts from people to help, such as my communication partner telling me that I don’t have to type to talk when the other person has left the conversation, or my support person telling me that I’ve finished my activity. [P123]

External prompts were appreciated when they ‘helped me get started, or helped me break down the tasks so that there were fewer barriers’ [P131] or when ‘someone has brought me out of it because they know I need to have something to eat and look after myself’ [P139]. However, external prompts could also be unpleasant – ‘I never react well to a sudden lurch from these states’ [P136]. In particular, ‘being told to come back’ [P109] while immersed in a task could cause participants to feel ‘frustrated that I can’t finish’ [P143] and ‘confused’ [P143, P115]. Participants described the experience of being interrupted as feeling ‘really jarring’ [P139], ‘like suddenly being woken from the deepest sleep’ [P136] or ‘like someone grabbed your shoulders and pushed you to a stop’ [P143]. Getting interrupted could also be taxing and lead to ‘a sudden loss in motivation’ [P143]:

Every change is costing me something in terms of my energy. If I’ve had interruptions, I’ll definitely be much more tired and I’ll probably lose the benefit of completing the task that I might have felt if I’d been able to finish it without interruption. [P143]

Although external prompting was sometimes unpleasant, it was also, at times, ‘essential – you sometimes have to be pulled out of these situations, or you could do them all day’ [P109].

Participants did not always want to be ‘pulled out of these situations’, however, as *being immersed in something could bring ‘a lot of joy’* (subtheme 4.3) – for example, while ‘doing something creative’ [P124], or while immersed in leisure activities, such as ‘cooking’ [P143], ‘gardening’ [P106], ‘reading, or even when I’m watching a TV series’ [P136]. Furthermore, participants found that ‘when I do stimming to de-stress, it becomes almost impossible to stop because of the calming effects’ [P123].

Participants found that they could be ‘incredibly productive . . . as I’m so engrossed’ and that ‘there’s something very satisfying about it while in the midst of it’ [P136]. These times could also bring ‘a sense of relaxation’ [P103] and make participants feel ‘physically kinda energised’ [P127]. Participants also experienced states described as being ‘the most amazing feeling in the world’ [P124], whereby they felt unable to stop because ‘my mind is in a state of flow’ [P113]. While in the ‘flow state’ [P142] or ‘hyper-focused state’ [P136], participants reported ‘exclusive focus . . . on that end goal to the exclusion of all else’ [P143], ‘forgetting to eat, and drink’ [136] and ‘losing all sense of time’ [P142]. These states would leave participants ‘on a bit of a high’ [P142]: ‘it feels wonderful’ [P113].

## Discussion

Here, we report a comprehensive investigation of Autistic people’s experiences of inertial rest and motion. We adopted an inductive (bottom-up) scientific approach, eliciting rich descriptions of Autistic people’s everyday experiences of ‘difficulties starting’ and ‘difficulties stopping’, and the broader context in which these phenomena occurred. Consistent with previous findings (Buckle et al., 2021; Phung et al., 2021; Welch et al., 2019, 2021), our participants described inertia as a pervasive and debilitating condition, whereby they struggled to transition between everyday tasks regardless of willpower.

Owing to these task-switching difficulties, participants’ day-to-day functioning across multiple important life domains was severely affected. What seemed to moderate the severity of these task-switching difficulties was the context; participants found it easier to manage their inertia in public settings (e.g. workplaces or community spaces) compared to private settings (e.g. home offices or private living spaces). In public settings, there were more likely to be external prompts that cued participants to switch tasks. This finding is in line with Buckle et al. (2021), who likewise found that Autistic adults relied heavily on external prompting to initiate tasks.

Our participants’ descriptions of inertia were not solely negative, however. They also described positive features, which stand in contrast to previous reports that have focused almost exclusively on the more disabling aspects (Buckle et al., 2021; Phung et al., 2021; Welch et al., 2019, 2021). Notably, our participants described how being immersed in a task could be productive and a source of joy. Some even described being unable to stop tasks because they had entered an optimal state of flow (although this could also have negative consequences). This study, therefore, extends previous findings by providing a more nuanced description of Autistic people’s experiences of inertia – the good and the bad (see also Rapaport et al., 2023, for further discussion).

Our interviewees’ report made it clear that Autistic inertia is a fundamental part of the Autistic experience. What is less clear, however, is whether inertia is also a uniquely Autistic experience. Although our participants described task-switching difficulties as part-and-parcel of being human, they differentiated the Autistic experience in terms of the intensity of these difficulties. Furthermore, our participants’ reports of difficulties starting tasks, regardless of willpower, suggest that this experience is distinct from the more common experience of procrastination, which is defined as a purposive delay in starting or completing tasks (Ferrari et al., 2005). Overall, these descriptions suggest that Autistic inertia may be both quantitatively and qualitatively different from the neurotypical experience of task-switching difficulties. Further research is needed to investigate this possibility, of course, perhaps by drawing Autistic participants into dialogue with non-Autistic participants who experience some elements of inertia to examine the (dis)similarities between these experiences.

Another possibility is that inertia is a transdiagnostic phenomenon. Many of our participants had co-occurring diagnoses of attention-deficit/hyperactivity disorder (ADHD), anxiety and depression (50%, 71% and 75%, respectively) – and recent reports of ‘ADHD inertia’ (task-switching difficulties as experienced by people with ADHD; Paul, 2019), as well as reports of feeling cognitively and emotionally stuck by people with depression and anxiety (Koval et al., 2012), suggest that inertia might indeed be experienced by people from across the neurodivergent community. Task immersion and flow experiences are not characteristic of depression or anxiety, however, suggesting that Autistic inertia and inertia associated with mental ill-health may be distinct phenomena. Whether ADHD inertia and Autistic inertia are related remains an intriguing question, however. The development of formal definitions will be necessary to determine how inertia might manifest across different neurodivergent profiles, and how these conditions might interact to give rise to inertial experiences.

Considering that inertia was described by our participants as being ‘the single most disabling part of being Autistic’ (also see Buckle et al., 2021), it is remarkable that this phenomenon has been overlooked in the academic literature, albeit with some recent exceptions (Buckle et al., 2021; Phung et al., 2021; Welch et al., 2019, 2021). One reason for this scarcity is that Autistic testimony has not always been taken seriously by researchers, but rather has been viewed as offering little more than anecdotal evidence (Jaswal & Akhtar, 2019; Pellicano et al., 2019). Learning from Autistic people is essential, especially if we are to understand what might be done to support Autistic people to overcome inertial challenges. Importantly, any support would need to be sufficiently targeted to reduce the disabling elements of inertia, while retaining and encouraging the positive elements.

Careful examination of first-person, subjective experiences should also directly inform more objective (‘third person’) scientific methods (e.g. Timmermann et al., 2023), including efforts to understand the mechanism(s) underlying inertial experiences. One possibility (also see Buckle et al., 2021) is that Autistic inertia may be underpinned by challenges with executive function – those cognitive processes essential for flexible, goal-directed behaviour in novel circumstances (Goldstein & Naglieri, 2014). Specific difficulties with cognitive flexibility and inhibition could give rise to challenges with stopping one task and switching to another. While difficulties with inhibition and flexible task switching could be experienced as distressing, the consequences could also be positive. Indeed, our participants described the joys of being unable to stop tasks because they were completely absorbed in a task. These reports are consistent with what Autistic scholars refer to as the ‘monotropic tendency’ (Lawson, 2011; Murray, 2018; Murray et al., 2005) – narrowly focused and sustained attention on a task of interest. It may be the case that the monotropic cognitive style gives rise to states of hyper-focus, both positive and negative. Difficulties with planning – another important executive component – might also explain aspects of Autistic inertia, including reports of difficulties identifying the first step of a task or planning purposeful motor movements through co-ordinating body parts. Nevertheless, executive function difficulties in Autistic people have been difficult to measure consistently (for reviews, see Demetriou et al., 2019; Hill, 2004; van de Cruys et al., 2014), possibly due to the weak ecological validity of traditional lab-based executive tasks (Burgess et al., 2006; Kenny et al., 2023; Kenworthy et al., 2008).

An alternative explanation of Autistic inertia is derived from a predictive coding account of autism (van de Cruys et al., 2013, 2014). Under predictive coding, perception is determined by both prior expectations about the world, as well as incoming sensory signals from the world (Clark, 2015; Hohwy, 2013; Rao & Ballard, 1999). Any mismatch between the expectation and sensory signal generates a prediction error. While some errors should be attended to, others are unreliable and should be ignored. For example, you might be more alert to a mismatch between what you *expected* your friend to say (‘hello’) and what you *heard* your friend say (‘yellow’) when in a quiet café (where the sensory input is more reliable), but the same mismatch might be ignored in a noisy café (where the auditory input is less reliable, and the error could be attributed to a mishearing).

However, the brains of Autistic people may treat *all* errors as salient (van de Cruys et al., 2013, 2014). Thus, in a volatile environment (e.g. a busy workplace or a cluttered home), an Autistic person might become overwhelmed by multiple, salient prediction errors, all competing for attention, leading to cognitive overload, and ultimately mental and physical paralysis (‘inertial rest’). In these situations, a prompt might help to discern which error signals are task-relevant and should be attended to and which to ignore. Alternatively, in response to excessive error signals, Autistic people might stim (‘inertial motion’) to flood the sensorium with a stream of easy-to-predict sensory signals that elicit fewer errors. This theory could therefore account for experiences of both inertial rest and motion within the same individual. This theory is also consistent with the descriptions of inertial rest being largely a negative experience, and inertial motion experiences being both good and bad.

Autistic people might also avoid volatile environments and instead seek out more-stable environments, routines or systems with rule-bound change. In more-stable contexts, matching a very precise prediction to a precise and stable sensory signal would generate less prediction error. It might therefore be appealing to remain immersed in stable, low-error contexts, rather than switching tasks and opening oneself to the possibility of encountering a high-error environment. Moreover, Autistic people might excel in stable environments where even a slight mismatch between a precise prediction and sensory signal would motivate learning. Actively reducing prediction errors may be intrinsically pleasurable, which could explain participants’ reports of flow state experiences, whereby Autistic people perform at the peak of their abilities.

The current findings cannot speak directly to either of these potential explanations of Autistic inertia. They nevertheless provide an important first step towards building a formal, community-driven definition of Autistic inertia. A formal definition is essential for understanding the causes of inertia and should allow researchers to develop tools for measuring and managing it.

### Limitations

Our study is not without its limitations. Our sampling and recruitment methods may have restricted the nature of our sample. For example, our participants were predominantly of white ethnic backgrounds and were more likely to identify as women, be highly educated, currently employed and communicate using spoken language. Although we offered a range of formats for the interview, its highly verbal nature may have discouraged or excluded Autistic adults who do not have strong written or spoken communication from participating. Future studies could employ maximum variation sampling (Patton, 1990) – purposively recruiting participants with very different backgrounds and life experiences – to ensure that the sample reflects the diversity of people within the Autistic community. It will also be important to develop innovative methods to understand how inertia manifests in Autistic people with intellectual disability and/or who are non- or minimally-speaking.

## Conclusion

In sum, our findings revealed that Autistic inertia is a double-edged sword, yielding both joyful and highly disabling experiences. These findings are an essential first step in developing a formal definition of Autistic inertia which will, in turn, be essential for raising awareness of Autistic inertia and reducing the stigma associated with inertial challenges, developing supports to manage these challenges and celebrating the unique advantages that Autistic inertia confers.

## Supplemental Material

sj-docx-1-aut-10.1177_13623613231198916 – Supplemental material for ‘I live in extremes’: A qualitative investigation of Autistic adults’ experiences of inertial rest and motion Supplemental material, sj-docx-1-aut-10.1177_13623613231198916 for ‘I live in extremes’: A qualitative investigation of Autistic adults’ experiences of inertial rest and motion by Hannah Rapaport, Hayley Clapham, Jon Adams, Wenn Lawson, Kaśka Porayska-Pomsta and Elizabeth Pellicano in Autism

## Notes

1. In the Autistic community, identity-first language (‘Autistic person’) is often preferred to, and considered less stigmatising than, person-first language (‘person with autism’) (Bottema-Beutel et al., 2021; Kenny et al., 2016) and is the preference of our Autistic co-authors. We therefore use identity-first language throughout. We have also chosen to capitalise the word Autistic to indicate a proper adjective, which reflects its status as a robust and valued identity and shared community, similar to the Deaf community.
