# Supplementary material for: ‘I live in extremes’: A qualitative investigation of Autistic adults’ experiences of inertial rest and motion
Source: Autism. 2023 Sep 30;28(5):1305–15. doi: 10.1177/13623613231198916 (PMC11067417; doi:10.1177/13623613231198916)
Supplement: sj-docx-1-aut-10.1177_13623613231198916 – Supplemental material for ‘I live in extremes’: A qualitative investigation of Autistic adults’ experiences of inertial rest and motion [file sj-docx-1-aut-10.1177_13623613231198916.docx]

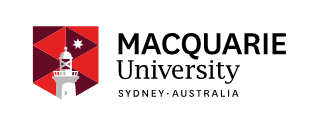


Understanding Autistic Inertia Semi-Structured Interview Schedule

**Introduction** *(script to be said by the interviewer):*

Hi [insert interviewee’s name], It’s great to talk with you today!

My name’s [insert interviewer name] and I’m a member of the Macquarie University autism research team.

Thank you for agreeing to help out with this research.

During our conversation today, I’d like to ask you about some of your life experiences—specifically, times in your life when you’ve experienced difficulties getting started with a task or activity,

as well as times when you’ve felt so immersed in a task, or thought pattern, that you couldn’t, or didn’t want to stop.

I sent you some preparation cards so you could have some time to think about these types of experiences in your life. Did you have a chance to look at those?

[Check the level of preparedness. If they didn’t have a chance, go through the interview preparation cards briefly and answer any questions they might have.]

Before we get started, I’d like to let you know a few things about our conversation today. Firstly, please feel free to answer at your own pace. We’re in no rush.

Secondly, you can skip a question or come back to a question at any time during our interview. You can also ask me to repeat or re-word a question at any time.

Sometimes I might ask you to say more about what you’ve just said so that I can get a clearer understanding of what you’re describing.

Is there anything you’d like to ask or have me clarify so far? [Wait for a response. Answer any questions]

Let me know if at any time you decide that you would like to stop the interview, move on to the next question, or take a break.

We’ve already talked about ways that you can let me know that you would like to stop, move on or take a break, by: [list ways].

Is it OK with you if I make a voice recording of our conversation? I ask because I won’t be able to write down everything you say. I will delete the recording once I have typed up your responses. And just to clarify, this would only be a voice recording and not a video recording. Is that ok?

Do you have any questions, before we start? [Answer any questions]

**Semi-structured interview questions:**

*Note. Semi-structured interviews include a short list of ‘guiding’ questions (1., 2., 3.,…) that are supplemented by follow-up and probing or follow-up questions (a., b., c.,…), which will only be asked if they have not already been covered by the interviewee’s responses. All questions should be open-ended, neutral and clear, and avoid leading language.*

| **Inertial rest** | | |
| --- | --- | --- |
| **Theme** | **Guiding questions** | **Probing questions** |
| Describe inertial rest | 1. To start, I’d like to ask you about a time in your life when you’ve found it difficult to get started on something – a task or an activity. [Pause] Please describe an experience like this to me. | 1. *What [task/*activity] were you trying to do at the time? 2. *What happened* when you tried to do the task? 3. Is this the *only time* this has happened to you? 4. Do you have other examples of these types of experiences? 5. Have there been other times when you have experienced this difficulty? 6. Does it *always* happen in *this way*? 7. Does it always happen in this *context*? 8. Does it happen in other contexts too? If so, please give me some examples. |
| Emotional response | 2. How do you feel when you experience difficulty getting started on a task? | 1. How do you feel physically when you can’t get started? 2. Do you notice any physical sensations in your body when this happens? 3. How do you feel emotionally during these experiences? 4. Are you able to identify any emotions that you feel during these experiences? 5. Can you describe the thoughts that you have when this happens? 6. Do you feel good, bad or neutral when this   happens? |
| Frequency | 3. How often do you experience difficulties getting started on a task? | a) Does it happen more than once a ? [Day / week / month] |
| Duration | 4. When you experience difficulties getting started on tasks, how long do these  experiences usually last? | a) Does it last ?  [A few minutes / a few hours / all day] |

| ***Would you like to keep going, or take a short break?*** | | |
| --- | --- | --- |
| Perceived cause(s) | 5. What do you think causes difficulties with getting started on tasks? | 1. Do you think the difficulty is caused by something in your mind? 2. Do you think the difficulty is caused by something in your body/muscles? 3. Do you think the difficulty is caused by both   your mind and your muscles? |
| Catalyst for change in state | 6. What eventually helps you to get started on a task after you’ve experienced this difficulty? | 1. Is there something that you think about that helps you? 2. Is there something that you do that helps you? 3. Is there something that someone else does or says that helps? 4. Is there a service or support system that helps   you? |
| Impact | 7. How do these difficulties with not being able to start tasks impact your life? | 1. Does it impact you in a good way? If so, how? 2. Does it impact you in a not-so-good way? If so, how? 3. Does it impact others in your life? |
| Other | 8. Is there anything else you would like to add about your experience of having difficulty with getting  started? |  |
| ***We’re about halfway through the session now.***  ***Would you like to keep going, or take a short break before doing the second half*?** | | |

| **Inertial Motion** | | |
| --- | --- | --- |
| **Theme** | **Guiding questions** | **Probing questions** |
| Describe inertial motion | 9. Now think about a time in your life when you’ve felt so immersed in a task or thought that you couldn’t (or didn’t want to) stop. Please describe an experience like this to me. | 1. Did you feel like you wanted to stop but couldn’t? Or that you didn’t want to stop? 2. What were you doing (or thinking about) at the time? 3. Did you feel as if you couldn’t stop, or didn’t want to stop? 4. *What happened* when you tried to stop? 5. Is this the *only time* this has happened to you? 6. Have there been other times when you have experienced this inability to stop? 7. Does it *always* happen in *this way*? 8. Does it always happen in this *context*? 9. Does it happen in other contexts too? If so, which ones? |
| Emotional response | 10. How do you feel when you can’t (or don’t want to*) stop a particular task or thought?  * Can’t vs don’t want to depends on response to the first ‘inertial motion’ question. | 1. How do you feel physically during these experiences? 2. Do you notice any physical sensations in your body when this happens? 3. How do you feel emotionally during these experiences? 4. Are you able to identify any emotions that you feel during these experiences? 5. Do you feel good, bad or neutral during these   experiences? |
| Frequency | 11. How often do you experience not being able to, or not wanting to stop a  task or thought? | a) Does it happen more than once a ? [Day / week / month] |
| Duration | 12. When you are unable to (or don’t want to) stop a task or thought, how long does  this experience usually last? | b) Does it last for ?  [A few minutes / a few hours / all day] |
| *Would you like to keep going, or take a short break?* | | |
| Perceived cause(s) | 13. What do you think causes you to be unable to (or unwilling to) stop a task or thought? | 1. Do you think the experience is caused by something in your mind/brain? 2. Do you think the experience is caused by something in your body/muscles?   Do you think the experience is caused by both  your mind/brain and your muscles? |

| Catalyst for change in state | 14. What do you think causes you to eventually stop the task or thought pattern? | 1. Is there something that you *think* about that causes you to stop? 2. Is there something that you *do that* causes you to stop?   Is there something that *someone else* does that causes you to stop? |
| --- | --- | --- |
| Impact | 15. How does being unable to (or not wanting to) stop a task or a thought impact  your life? | 1. Does it impact you in a good way? If so, how? 2. Does it impact you in a not-so-good way? If so, how? 3. Does it impact others in your life? |
| Other | 16. Is there anything else you would like to add about your experience of having difficulty with getting started? |  |
| **Final questions** | | |
| Other | 17. Is there anything else that you would like to say about the topics that  we’ve discussed today? |  |
| Support | 18. Did you receive any  support to complete this interview? | a) If so, can you describe the support that you received? |

**Ending the interview** *(script to be said by the interviewer):*

*Thanks [insert interviewee name], that’s all my questions!*

*I really appreciate the time you’ve taken to share your experiences with me. In about a month, I will send you a typed-up version of this conversion.*

*When you receive it, you can look through the document and make sure that you’re comfortable with the information that you’ve shared with me.*

*If there are parts of this conversation that, for whatever reason, you would like to be removed, you can let me know and I will delete those bits!*

*I hope that you enjoyed our conversation, but if you feel worried or upset as a result of what we’ve spoken about today, please get in touch with your support person.*

*I’ve also included the phone numbers for Beyond Blue and Lifeline in your Support Person letter, in case you’d like to speak to someone anonymously about any concerns you have following this interview.*

*Thank you so much again and I look forward to being in touch again soon.*
